# A novel p.127Val>Ile single nucleotide polymorphism in the *MTNR1A* gene and its relation to litter size in Thin-tailed Indonesian ewes

**DOI:** 10.5713/ab.24.0187

**Published:** 2024-06-25

**Authors:** Mutasem Abuzahra, Mohammed Baqur S. Al-Shuhaib, Dwi Wijayanti, Mustofa Helmi Effendi, Imam Mustofa, Ikechukwu Benjamin Moses

**Affiliations:** 1 Doctoral Program in Veterinary Science, Faculty of Veterinary Medicine, Universitas Airlangga, Surabaya 60115, Indonesia; 2 Department of Animal Production, College of Agriculture, Al-Qasim Green University, Al-Qasim, 51013, Babil, Iraq; 3 College of Animal Science and Technology, Northwest A&F University, Yangling, Shaanxi, 712100, China; 4 Department of Veterinary Public Health, Faculty of Veterinary Medicine, Universitas Airlangga, Surabaya 60115, Indonesia; 5 Department of Veterinary Reproduction, Faculty of Veterinary Medicine, Airlangga University, Surabaya 60115, Indonesia; 6 Department of Applied Microbiology, Faculty of Science, Ebonyi State University, Abakaliki 481101, Nigeria

**Keywords:** Genetic Diversity and Farmed Animals, Litter Size, *MTNR1A*, Single Nucleotide Polymorphisms

## Abstract

**Objective:**

The primary objective was to identify and characterize the single nucleotide polymorphisms (SNPs) within the *MTNR1A* gene sequence in Thin-tailed Indonesian ewes to assess the possible association of *MTNR1A* gene polymorphism with litter size trait.

**Methods:**

Forty-seven Thin-tailed Indonesian sheep were selected for the study. Genotyping involved collecting blood samples, and sequencing exon 2 of the *MTNR1A* gene.

**Results:**

The study identified 19 novel SNPs, with 10 being non-synonymous variations, in the *MTNR1A* gene of Thin-tailed Indonesian ewes. One non-synonymous SNP (rs1087815963) showed a significant association with litter size, with the GC genotype exhibiting a higher average litter size than the GG genotype. The deleterious impact of p.Val127Ile SNP was predicted by various *in silico* tools that predicted a highly damaging effect of p.Val127Ile SNP on the structure, function, and stability of MTNR1A. Docking reactions showed a critical involvement of this locus with the binding with melatonin.

**Conclusion:**

In conclusion, the results of our study suggest that rs1087815963 has a remarkable negative impact on the MTNR1A with a putative alteration in the binding with melatonin. Therefore, the implementation of the novel p.Val127Ile could be a useful marker in marker-assisted selection.

## INTRODUCTION

In 2022, the sheep population in Indonesia was recorded at 15.62 million head, showing a slight decrease of 0.13% compared to the previous year’s count of 15.64 million sheep. This is a notable shift from the peak population of 17.77 million sheep in 2020. Interestingly, West Java province contributes significantly to Indonesia’s sheep population, housing approximately 12.27 million sheep, which constitutes a substantial 69% of the nation’s total sheep population [1]. Sheep reproduction is an essential economic characteristic [2–4], The complicated trait affecting multiple indicators such as fertility, fecundity, and prolificacy [5], is a complicated trait with low (5% to 10%) heritability that is influenced by both genetic and environmental [6]. Identifying candidate genes and their causative mutations is a powerful method for comprehending the genetic process that contributes to the diversity in reproductive performance in sheep [2].

In sheep breeding, litter size is the primary limiting factor, controlled by numerous unidentified factors. The progress in science and technology has enabled the utilization of molecular genetics and biology advancements to enhance the number of offspring produced [7]. In order to expedite the reproductive rate and diversify agricultural sectors, efforts are being made to enhance the breeding speed. This will enable the production of high-quality economic animal products to fulfill the demands of a rapidly growing population [8]. The investigation of genes associated with litter size, whether through direct or indirect means, has gained popularity in recent decades. Genome-wide association studies (GWAS) are being utilized more and more to investigate the candidate genes that have polymorphisms associated with a certain phenotype [9,10], and marker-assisted selection (MAS) enables precise and rapid analysis of the genetic makeup of individuals at the molecular level, facilitating the selection of specific genotypes. Due to the substantial decrease in the cost of genetic marker detection, it is now feasible to simultaneously examine the presence of various markers in one individual [7]. Traditional selection approaches should be complemented with MAS to augment reproductive capability. Utilizing MAS for the validation of candidate genes will significantly enhance breeding efficiency [11]. The reproductive seasonality of ewes is characterized by the presence of a seasonal anestrus period, which refers to the number of days from when rams are introduced to ewes until lambing occurs. This time is regulated by the photoperiod [12].

Melatonin governs the seasonal reproductive processes in mammals by regulating the secretion of follicle-stimulating hormone and luteinizing hormone via the hypothalamic-pituitary-gonadal axis [13]. Melatonin (MT) is a hormone derived from indole that is produced in the pineal gland [14,15]. The melatonin receptor (MTNR) plays a crucial role in various biological processes such as the control of animal sexual behavior, reproduction, and circadian rhythm [16,17]. The melatonin receptors can be classified into two subtypes: melatonin receptor subtype 1A (*MNTR1A*) and melatonin receptor subtype 1B (*MTNR1B*) [14,18].

*MTNR1A* is primarily localized in the suprachiasmatic nucleus and pituitary nodules within the hypothalamus of mammals. Its function is closely associated with the regulation of animal reproduction [14]. He at al [19] discovered that administering melatonin before the peak expression of *MTNR1A*, which occurs before ovulation, can enhance luteal function, elevate progesterone secretion levels, and improve the pregnancy rate and litter size of mice. Various studies have discovered connections between *MTNR1A* and reproductive functions in various animal species [17]. As of now, a considerable number of studies have delved into the association between polymorphisms of MTNRs and traits related to litter size or reproductive seasonality in various mammalian species [6,20,21]. Studies in sheep have predominantly concentrated on the sites 606 and 612 within the *MTNR1A* gene exon 2. Mutations identified at these positions have been suggested as potential contributors to seasonal reproduction [22,23].

The Rasa Aragonesa breed has been found to have an association between the polymorphism SNP rs403212791 and reproductive seasonality [16]. In Barbarine ewes, the study identified two single nucleotide polymorphisms (SNPs), namely rs430181568 and rs40738822721, demonstrating complete linkage and a robust correlation with the resumption of reproductive activity. Significantly, these SNPs were found to exert a substantial impact on the birth weights of lambs. Specifically, Barbarine ewes with the A/A genotype at these SNPs exhibited elevated birth weights, implying a potential influence on reproductive efficiency [12].

Therefore, a multitude of studies has scrutinized the association between *MTNR1A* and reproductive traits across diverse species, designating it as a potential candidate gene for quantitative trait loci. Nonetheless, findings in sheep underscore that the relationship between *MTNR1A* gene polymorphism and reproduction can exhibit variability based on the breed [17]. In the *MTNR1A* gene exon II, different sheep breeds exhibit nucleotide variations. These are assumed to change the reproductive response to seasonal variations and to improve, in general, the reproductive performance [24] Given these considerations, the objective of this study is to identify and characterize the patterns of SNPs within the *MTNR1A* gene sequence, and to evaluate the possible association of these SNPs with litter size traits in Thin-tailed Indonesian ewes. Additional assessment of the potential molecular mechanism by which the identified SNPs affect litter size is deduced using various computational tools. The utilization of in silico prediction has enhanced the classical genotype-phenotype association by offering a possible three-dimensional elucidation for the variations in performance between the normal and altered MTNR1A protein in ewes with wild-type and altered alleles, respectively.

## MATERIALS AND METHODS

### Ethical approval

The Animal Care and Use Committee (ACUC) under the control of the Faculty of Veterinary Medicine at Airlangga University approved the use of animals in experiments and granted ethical permission for this study (No: 1.KEH.117.09. 2022). Furthermore, the experimentation on animals was conducted in strict adherence to the pertinent local legislation and regulations governing animal care.

### Animals and management

A total of 47 Thin-tailed Indonesian ewes were used in this study. The animals were raised and located in the East Java Island of Indonesia (Malang); the farm is located at 8.0553° S, 112.5066° E near Kawi Mount. In the study, the animals were provided with leguminous and gramineous grasses equivalent to 10% of their body weight during the day. Additionally, they were given a daily ration of commercial concentrate feed, amounting to 5% of their body weight per head. The concentrate feed had a composition of 15% crude protein.

### Genotyping

A blood sample was obtained from each sheep by collecting it from the jugular vein using a sterile vacuum tube (BD Vacutainer System, Believer Industrial Estate, Plymouth, UK) using ethylenediaminetetraacetic acid as an anticoagulant. Afterward, 5 mL were taken from each sample and kept at a temperature of −20°C until further analysis. The primers employed for amplifying the exon 2 region, spanning 824 bp (F: 5′-TGT GTT TGT GGT GAG CCT GG-3′ and R: 5′-ATG GAG AGG GTT TGC GTT TA-3′) in the *MNTR1A* gene were the ones documented by Saxena et al [ 25]. Polymerase chain reaction (PCR) was conducted using the gradient PCR system T100 Thermal Cycler (Bio–Rad, Hercules, CA, USA) with a reaction volume of 30 μL. The reaction mix consisted of 2.5 μL genomic DNA, 0.5 μL each of forward and reverse primer, 12.5 μL *Taq* Green PCR Master Mix, and 14-μL ddH2O. The PCR amplification was conducted according to the following protocol: an initial denaturation step at 94°C for 3 minutes, followed by 35 cycles of denaturation at 94°C for 1 minute, annealing at 57.7°C for 45 seconds, and elongation at 72°C for 1 minute. The amplification was completed with a final extension step at 72°C for 10 minutes. The expected lengths of PCR products were verified by agarose gel electrophoresis (1.5% W/V) using ethidium bromide as a staining dye. Subsequently, the 25 μL PCR result was sent to a commercial laboratory service (1st BASE Laboratories Sdn Bhd, Selangor, Malaysia) for sequencing analysis using Sanger DNA sequencing with capillary electrophoresis. The DNA sequences were examined utilizing the BioEdit tool version 7.00, developed by Tom Hall of Ibis Therapeutics in California, USA. The SNPs were detected by comparing them to the reference genomic sequences of the *MTNR1A* gene. The detected SNPs were confirmed by reviewing their original electropherogram files. The variant effect predictor (VEP), a tool provided by the Ensembl genome browser, was utilized to forecast the consequences of a missense variant on the structure and function of a protein. The VEP can be accessed at https://www.ensembl.org/Ovisaries/Tools/VEP?db=core.

### Statistical analysis

Genotyping data were calculated using Pop Gene version 1.32 to compute allele and genotype frequencies, assess polymorphism information content (PIC), evaluate heterozygosity (HE), determine the number of effective alleles, and use chi-square (χ^2^) tests to derive the corresponding p-value. Based on the identified genetic polymorphism of the *MTNR1A* gene, the PIC is categorized into three levels: high (PIC>0.5), intermediate (0.25<PIC<0.5), and low genetic diversity (PIC <0.25) [26]. An analysis of variance was conducted using IBM SPSS 24.0 software to analyze the association between litter size and genotypes. *Y_ij_* = *μ*+*G_i_*+*e_ij_*, where *Y_ij_* is the litter size phenotype of the individual Thin-Tailed sheep, *μ* is the population mean, *G_i_* is the effect of genotype or haplotype, and *e_ij_* is the random error effect. The continuous variables were represented as mean±standard deviation (SD) and p< 0.05 was considered to be significant. Using the Haploview v4.2 software [27], the linkage disequilibrium (LD) was evaluated among genotypes, and the structure of LD was determined using the D’ and r^2^ parameters. If r^2^>0.33, the LD is considered as sufficiently strong; and if r^2^ = 1, the LD is complete.

### Prediction of protein interaction with substrate

The amino acid sequence of the MTNR1A protein in sheep was obtained from the NCBI database (protein ID: NP_001 009725.1). To assess the impact of non-synonymous SNPs (nsSNPs) on the MTNR1A protein, several analyses were conducted. Firstly, the SIFT tool was used to predict whether the nsSNPs were deleterious or non-deleterious [28], Additionally, the PANTHER tool was employed to determine the potential functional impact of these nsSNPs [29]. The results obtained from SIFT and PANTHER were further validated using the PolyPhen-2 tool [30]. The stability of the protein following mutation was assessed using the I-Mutant2 tool [31]. Since there was no crystallized structure of MTNR1A in the Protein Data Bank (PDB) server (https://www.rcsb.org/), the 3D structure of the protein was generated using the Swiss Model tool (https://swissmodel.expasy.org/interactive). The validity of the 3D structure was confirmed using the Ramachandran plot, and the overall model quality was assessed using the ProSA web server (https://prosa.services.came.sbg.ac.at/prosa.php). To describe the status of each amino acid before and after the missense variation within the 3D structure of the protein, PyMol-v1 from Schrödinger, LLC was used. Furthermore, predictions of the effect of nsSNPs on the 3D structure of the altered protein were performed using mSCM [32] and MUpro [33] prediction tools.

### Docking with melatonin

To comprehensively predict the effect of the identified nsSNPs on the binding of MTNR1A with melatonin, two separate molecular docking reactions were performed. These reactions aimed to assess the binding efficiency of melatonin with both the normal and altered forms of MTNR1A. Before conducting the docking with MTNR1A, the 3D conformer of melatonin (C13H16N2O2) was retrieved from the PubChem server (PubChem CID 896) in SDF format. The blind docking was carried out using the CB-Dock tool with its default settings [34]. Melatonin and MTNR1A were docked, and the docking scores and cavity sizes for the best pose binding of the normal and altered MTNR1A with melatonin were visualized and compared using the PyMol tool. Additionally, annotations for the bonds shared between the interacting amino acids with melatonin were provided by the protein-ligand interaction profiler (PLIP) [35].

## RESULTS

### SNPs identification

A total of 824 base pairs (bp) were sequenced from the targeted exon 2 locus. The patterns of the genetic variations were determined using a comparative analysis of ovine sequences, namely GenBank sequence NM_001009752.1 analysis of sequences obtained from the entire population (n = 47) identified a total of 19 genetic variations (Table 1). Table 1 displays the SNPs’ location, alias (nomenclature used in this manuscript), dbSNP identifiers, amino acid substitution effect. The *MTNR1A* gene is located in the reverse orientation of the genome. The SNPs are arranged based on their position in the most recent version of the genome (Oar4.0: GenBank accession number NW_014639035.1. In exon 2, ten non-synonymous and nine synonymous SNPs were identified in exon 2. Among the analyzed SNPs, five were newly discovered, with four causing changes in the amino acid and one resulting in a premature stop codon.

### Population genetic analysis of polymorphism in the *MTNR1A* gene

Population genetic analysis was performed to investigate 19 loci of the Thin-tailed Indonesian sheep breed. The findings indicate that the snp1, snp6, snp7, snp9, snp12-snp14, and snp16-snp18 loci exhibited a moderate level of polymorphism (0.25<PIC<0.5) across all sheep breeds. On the other hand, the snp-snp5, snp8, snp10, snp11, snp15, and snp19 loci exhibited a low level of polymorphism (PIC<0.25) in loci (as shown in Table 2). The SNPs that were analyzed most often in previous studies on a variety of sheep breeds were rs406779174, rs430181568, rs407388227, and rs403212791. In our analysis, we discovered that only SNP rs407388227 did not result in Hardy–Weinberg equilibrium (p<0.05).

### Association analysis of *MTNR1A* gene with litter size in Thin-tailed Indonesian ewes

A study was conducted to analyze the association between the 19 polymorphic loci and litter size of Thin-tailed Indonesian sheep. The table presents the least squares means and SDs of average litter size. There was a statistically significant association between non-synonymous snp2 (rs1087815963) and litter size in Thin-tailed sheep, with ewes with the GC genotype producing a larger litter size than those with the GG genotype. Another significant association was identified between synonymous snp11 (rs420819884) and litter size, as sheep with the AG genotype showed larger litter size than those with the GG genotype. The remaining loci were not significantly associated with litter size (Table 3).

### Linkage disequilibrium estimation

**Figure 1:**
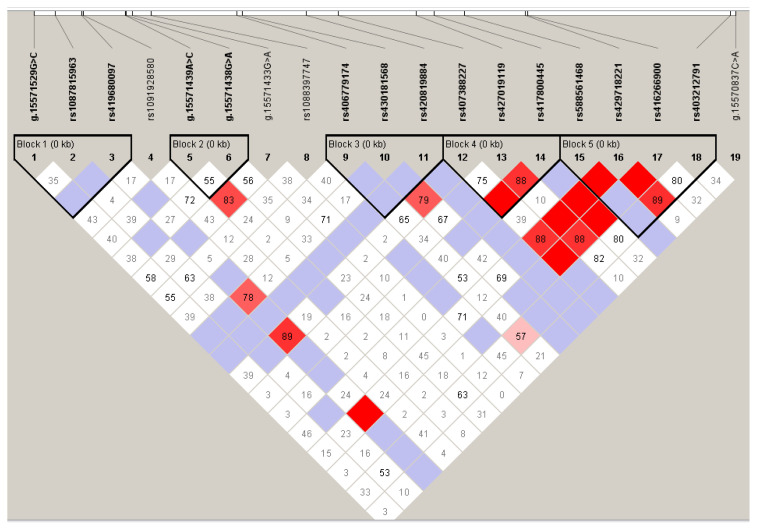
Linkage disequilibrium among the nine single nucleotide polymorphisms of the *MTNR1A* gene in Thin Tailed Indonesian sheep is evident. The color of the squares corresponds to the degree of linkage, where darker shades indicate higher levels of linkage; The numerical values within the squares represent the strength of the correlation between locations, expressed as a percentage. Haplotype blocks are indicated by dark lines.

To examine the possible connections between different variations within the *MTNR1A* gene, we performed a thorough study of LD using Haploview4.2 software, as illustrated in Figure 1. LD analysis revealed a notable scarcity of potential co-inheritance with neighboring SNPs within the genomic block where rs1087815963 SNP is situated. The results of our study showed that there is a significant association between 15 SNPs, which may be grouped into five separate blocks. The SNP rs427019119 and rs417800445 (snp13 and snp14 in our study, respectively) had a strong linkage relationship (D′ = 0.888and r^2^ = 0.789), as shown in Table 4 allowing us to classify them as a single marker. The LD between snp12 and snp3 exhibited long-range association (D′ = 0.89 and r^2^ = 0.512). Employing a 5% haplotype frequency threshold in Haploview4.2, we identified a total of 18 haplotypes across the 15 SNPs within the Thin-tailed Indonesian ewe *MTNR1A* gene.

### Haplotypes frequency and association analysis of *MTNR1A* gene haplotypes and litter size in Thin tailed Indonesian ewes

The correlation between haplotypes of the *MTNR1A* gene and litter size in Thin-tailed Indonesian ewes was examined, and the findings are summarized in Table 5. The data revealed a statistically significant association between the four haplotypes in block 1 (snp1, snp2, and snp3) and litter size, with a p<0.05. Haplotype H1 (AG) in block 2 had the highest frequency among the 18 mentioned haplotypes, with a frequency of 0.727.

### *In silico* predictions

Out of ten non-synonymous SNPs, only one (rs1087815963) exhibited a significant association with litter size in the investigated ovine population. Due to its significant association with these traits, the possible effect of this SNP on the biological activity of MTNR1A was explored. Four *in silico* tools could not individually predict the effect of the missense SNPs, whether neutral or harmful [36]. Therefore, the combined utilization of numerous computational algorithms was applied to predict the effect of the detected SNPs. The effect of the detected missense SNPs in MTNR1A on structure, function, and stability was analyzed using a set of six different state-of-the-art in silico tools (SIFT, PANTHER, PolyPhen-2, I-Mutant2, mCSM, and MUpro). These computational analyses predicted a highly deleterious effect of p.127Val>Ile on the MTNR1A (Figure 1). The cumulative prediction of these tools suggested a highly putative damaging role for this nsSNP in the scheduled MTNR1A-based biological activities in ewes with GC genotype. In contrast, none of the other non-synonymous changes were anticipated to be detrimental; all were classified as tolerated based on the absence of a finding in at least four of these tools. The SIFT scores for the SNPs are as follows: The values for snp17, snp18, and snp12 are 0.51, 0.29, and 0.23, respectively.

To obtain further details of the putative mode of action of the identified missense p.127Val>Ile variant on the MTNR1A, a 3D structure was generated and its quality was assessed. Ramachandran plot showed that the generated model was in a highly qualified physiochemical characteristics since more 97.25% of the amino acid residues were resided in the Ramachandran favoured region (Supplementary File 1). ProSA web showed that the generated PDB model was similar to the native models of similar sizes (Supplementary File 2).

### Docking outputs

**Figure 3:**
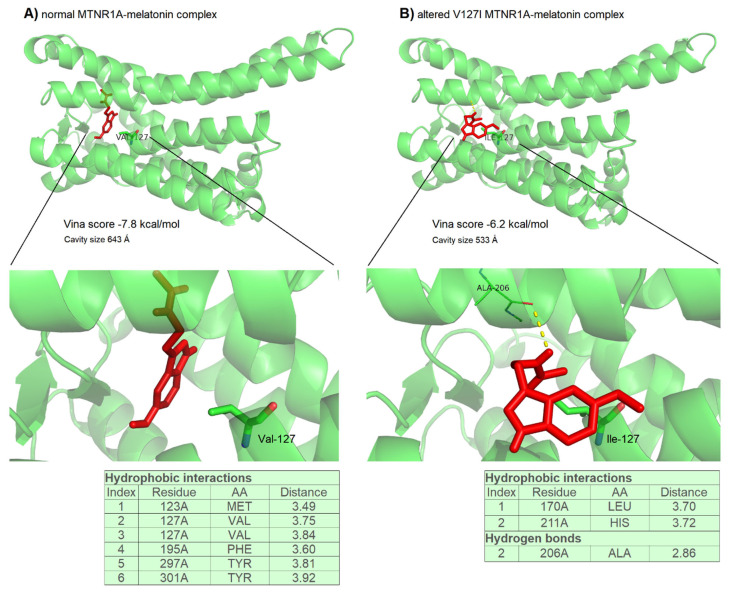
Docking between MTNR1A with melatonin. (A) Normal MTNR1-melatonin complex, (B) Altered (p.127Val>Ile) MTNR1-melatonin complex. Melatonin is shown as a red stick, while Val127 and Ile127 are shown as green sticks.

To evaluate the damaging impact of the p.127Val>Ile variant on MTNR1A and its effect on the binding of the receptor to melatonin, two docking tests were conducted. These tests aimed to assess the efficacy of the binding with melatonin before and after the mutation. Since MTNR1A plays a role in regulating reproductive metabolism and the circadian action of melatonin, understanding the impact of this variant is crucial for elucidating its functional consequences. The first docking was carried out between the wild-type MTNR1A and melatonin, and the second docking was conducted between the altered MTNR1A and melatonin under the same conditions. The docking score of the wild-type MTNR1A-melatonin binding was −7.8 kcal/mol, while the cavity size was 643 Å (Figure 3a). PLIP analysis revealed that the docking score of −7.8 kcal/mol for the wild-type MTNR1A-melatonin binding was generated from the involvement of five amino acid residues in the interaction with melatonin, including Val127, which contributed two hydrophobic interactions (Figure 3a). In the second docking, the altered MTNR1A showed a notable alteration in the binding architecture with melatonin, resulting in a significant reduction in binding efficacy upon the p.127Val>Ile mutation. The docking score for the mutant MTNR1A-melatonin interaction decreased to −6.2 kcal/mol (with a cavity size of 533 Å), with only two amino acid residues participating in the hydrophobic interaction with melatonin. In contrast to the wild-type MTNR1A protein, the altered receptor exhibited largely reduced hydrophobic interactions with melatonin, which was excluded by only two shared with Leu170 and His210. Meanwhile, the altered receptor had shared one H-bond with melatonin using Ala206 residue (Figure 3b).

## DISCUSSION

One of the essential goals of breeding is to detect genetic variation that affects highly beneficial traits, such as fertility. This attribute significantly influences the overall profitability of the sheep industry. The capacity to control genetic variation holds the promise to augment breeding efforts, especially if DNA MAS can be utilized to more consistently, expeditiously, and economically identify superior animals. It is advantageous for a quality or characteristic to only appear once an individual achieves maturity, as is the case with reproduction.

In our extensive sequencing analysis of the *MTNR1A* gene in Thin-tailed Indonesian sheep, we discovered 19 SNPs that were specifically found in exon 2. It is worth mentioning that some SNPs identified in our study have been previously recorded in different breeds through prior research investigations [5,12,22,24,37–39] highlighting the extensive prevalence of these genetic variations. It is important to note that our inquiry has identified seven novel SNPs in exon 2. These SNPs are g.15571529G>C, rs108 7815963, rs1091928580, g.15571439A>C, rs1088397747, rs588561468, and g.15570837C>A. In our study, we have referred to these SNPs as snp1, snp2, snp4, snp5, snp8, snp15, and snp19. Remarkably, our research has successfully identified these specific genetic variants for the first time, making a significant addition to the comprehension of the genetic makeup of MTNR1A in Thin-tailed Indonesian sheep.

The Thin-tailed Indonesian ewes exhibited a notable genetic characteristic, specifically ten polymorphic sites that induced alterations in the amino acid sequence. This quantity surpassed the corresponding counts documented in various breeds, including Sarda [37], Aragonesa [16,24], Awassi [5], and Tunisian breeds Barbarine and Queue Fine de l’Ouest breeds [12]. The observed SNPs suggested a higher level of genetic variety in Thin-tailed Indonesian ewes, specifically in relation to the described polymorphic sites and their consequent effect on the composition of amino acids, as compared to the mentioned breeds. A study of the exon II sequence of Thin-tailed Indonesian ewes uncovered a consistent link between snp10 (rs430181568) and snp12 (rs407388227), which is consistent with similar discoveries in other breeds [23,39,40]. These findings suggest that either one or both SNPs may have a role in regulating the timing of reproductive cycles [5,39]. Though our investigation indicated that snp10 and snp12 as the most polymorphic loci, both SNPs did not exhibit any significant association with litter size in Thin-tailed Indonesian sheep. This observation aligns with the other similar findings in Awassi [5,12] and Istrian Pramenka ewes [38]. Interestingly, our results diverge from those reported by Vandeputte et al [41], where snp10 was associated with variations in litter size.

Individual SNPs may be directly used in research to investigate their correlation with phenotypic traits, but their impact on traits might be relatively small [42]. Linkage disequilibrium analysis emerges as a valuable method for probing the interconnected relationships among SNPs, as supported by numerous studies. Haplotypes encompass a greater amount of information compared to individual SNPs or the mere aggregation of numerous SNPs [43], the interactions between genetic loci, elucidated through such analysis, can provide a more accurate and comprehensive understanding of the genetic information underlying phenotypic traits. Haplotypes are superior to SNP loci as genetic markers due to their increased likelihood of being inherited in combination. Haplotypes shown to be more successful than SNPs for variables with moderate to low heritability [44]. According to Martínez-Royo et al [45], year-round estrus in Rasa Aragonesa sheep is linked to haplotypes of the 612 location T, A, and T alleles in exon 2 and the 422 and 677 positions in the *MTNR1A* gene promoter. Calvo et al [16] reported 9 SNPs in the *MTNR1A* gene promoter and 3 SNPs in exon 2 in Rasa Aragonesa sheep, further analysis of haplotypes indicated that the primary influence on seasonal reproduction is primarily due to non-synonymous alterations at position g.15099004G>A in exon 2. The current study, through haplotype analysis, reinforces our findings, particularly in Block 1 (comprising H1, H2, and H3). In this block, all haplotypes share the G allele at position 2, corresponding to snp2 (rs1087815963). Notably, Block 1 haplotypes are strongly associated with an increased litter size in Thin-tailed sheep, suggesting their significant role in enhancing reproductive performance. Furthermore, our results reveal a robust linkage between snp13 and snp14 (rs427019119 and rs417800445), as well as between snp16 and snp17 (rs429718221 and rs41 6266900). In contrast, the LD between snp12 (rs419680097) and snp3 (rs419680097) exhibited a long-range association. This extended LD can be attributed to intensive artificial selection in commercial breeding populations, leading to a reduction in effective population size. Additionally, LD varied significantly between chromosomes, indicating differences in autosomal recombination rates influenced by genetic drift and selection pressures within these populations [46]. This implies that these pairs of SNPs tend to be inherited together, potentially influencing specific genetic traits related to reproduction in Thin-tailed sheep. These strong linkages highlight potential genetic interactions contributing to the observed reproductive outcomes.

The current study revealed a significant correlation between a specific SNP, namely snp2 (rs1087815963), and litter size. The GC genotype exhibited a markedly greater average litter size (2.57±0.787) compared to the GG genotype (1.68 ±0.572). The snp2 variation, which we discovered throughout our analysis, is considered to be a novel finding. However, due to its possible weak collaboration with other surrounding SNPs, the LD findings suggest that the rs1087815963 SNP can largely be inherited independently. This suggests that its inheritance may occur irrespective of other SNPs. Accordingly, the finding of this SNP indicated that the rs1087815963 SNP may not participate with our SNPs in the determination of its impact on the metabolic pathway in which MTNR1A is involved.

The recent advancements in computational tools for predicting the effects of SNPs on protein structure and function have greatly facilitated data interpretation in animal breeding [47]. In our study, the utilization of these tools proved to be highly beneficial in elucidating the impact of observed SNPs on the MTNR1A protein in Thin-tailed Indonesian sheep. Specifically, the identification of the V127I mutation as deleterious by computational algorithms provided crucial insights into its potential effects on the structure, biological functions, and stability of MTNR1A. Multiple in silico affirmations of the deleterious effects of the V127I mutation further supported its potential to disrupt the normal functioning of MTNR1A. This mutation, occurring within a critical transmembrane helix region of the protein, may negatively influence its ability to interact with ligands or participate in signaling pathways crucial for reproductive processes in sheep. Considering the essential role of melatonin signaling, mediated by MTNR1A, in regulating seasonal reproduction and circadian rhythms [48], any disturbance in its function due to the V127I mutation could have significant implications for reproductive performance, including litter size traits.

This study employed molecular docking to understand how the p.127Val>Ile SNP modifies MTNR1A action by predicting a substitution of valine with isoleucine at position 127 (V127I) in the third transmembrane domain (TM3), crucial for ligand binding and receptor activation. Molecular docking indicated that the altered MTNR1A had reduced binding with melatonin, resulting in a smaller receptor-ligand cavity. These findings align with previous research on melatonin receptor mutations Chugunov et al [49] discussed how TM3 rotation in the MT2 receptor, involving Val124, affects ligand accessibility and interactions, similar to the p.127Val>Ile mutation effects observed here, which reduced binding efficacy and altered hydrophobic interactions with melatonin. Additionally, Calvo et al [50] highlighted the significance of specific amino acid residues like His195/208^5.46^, Ser110^3.35^, and Ser114^3.39^ in ligand binding and receptor activation, further emphasizing the critical role of TM domain residues in melatonin receptor function. Collectively, these insights underscore the importance of understanding how mutations in TM domains impact receptor-ligand interactions and signaling pathways. Due to the critical role played by MTNR1A in the binding with melatonin, any amino acid substitution that has a deleterious effect on its structure could impact this binding. When the function of this binding protein is impaired or reduced, it can disrupt the normal metabolism of melatonin and lead to variable effects on reproductive traits. When melatonin cannot be transported effectively in the body of sheep, it can disrupt various physiological processes regulated by melatonin, including reproductive functions. Due to the involvement of melatonin in the regulation of the estrous cycle and fertility, disturbances in the reproductive system with a potential decreased litter size result when it is not transported well. The predicted deleterious effects of the V127I mutation on MTNR1A activity underscore its relevance as a genetic marker for reproductive performance in Thin-tailed Indonesian sheep.

By providing insights into the biological activity of MTNR1A and its potential effects on litter size, our study contributes to a better understanding of the genetic basis of reproductive traits in sheep. The differences in how the melatonin signal is perceived could perhaps explain the observed variances in reproductive recovery phenotype in this investigation. In addition to our finding, it has been deduced that missense can have a substantial effect on complex characteristics since it has been demonstrated that point mutations in BMP15, MTNR1A, BMP7, and BMP2 have a substantial impact on litter size [24,51,52]. However, litter size is a complex process influenced by various factors, including diet, physical activity, genetics, and overall energy balance. The control of litter size is a multifactorial event, and it therefore cannot be easily determined. While our study in MTNR1A focused on the association between the V127I mutation and litter size traits in Thin-tailed Indonesian sheep, it is important to acknowledge the complexity of reproductive traits, which are influenced by multiple genetic and environmental factors. Thus, although the V127I mutation may represent a significant genetic determinant of litter size variation, it is likely just one piece of the broader genetic landscape governing reproductive outcomes in sheep. Furthermore, the high frequency of the V127I mutation in ewes-producing twins suggests its potential role as a causative factor for increased prolificacy in this population. It is well known that several loci contribute to the control of the ovulation rate in sheep [53]. Nevertheless, it is essential to consider that the impact of SNPs on the investigated phenotypic variables, particularly litter size, might have been influenced by the sample size. The need for a more extensive sample size is evident, and further research at a later stage is warranted to enhance our understanding of these genetic interactions and their implications for selective breeding strategy.

## CONCLUSION

Our study in Thin-tailed Indonesian ewes identified 19 SNPs in exon 2 of the *MTNR1A* gene. Among them, ten SNPs showed non-synonymous variations. Association analysis of litter size revealed a significant correlation with snp2 (rs1087815963), where ewes with the GC genotype displayed a higher litter size than those with the GG genotype. This novel finding was associated with a substitution (Val> Ile) at amino acid 127, which is crucial for MT1A receptor functionality. Molecular docking experiments revealed a decrease in MTNR1A binding when mutated with the p.127Val>Ile SNP. Reduced binding to melatonin suggests that this crucial hormone may not be adequately transported and utilized within cells. Consequently, more melatonin may accumulate in extracellular compartments, potentially resulting in various consequences, including a presumed decrease in litter size. Our findings suggest that the p.Val127Ile SNP in MTNR1A has dual successive effects: it negatively affects the biological activity of MTNR1A, which negatively influences reproductive performance by lowering litter size indices. Though p.127Val>Ile is strongly recommended as a crucial candidate for MAS in sheep, further *in vitro* experiments are required to validate this finding.

## Figures and Tables

**Figure 2:**
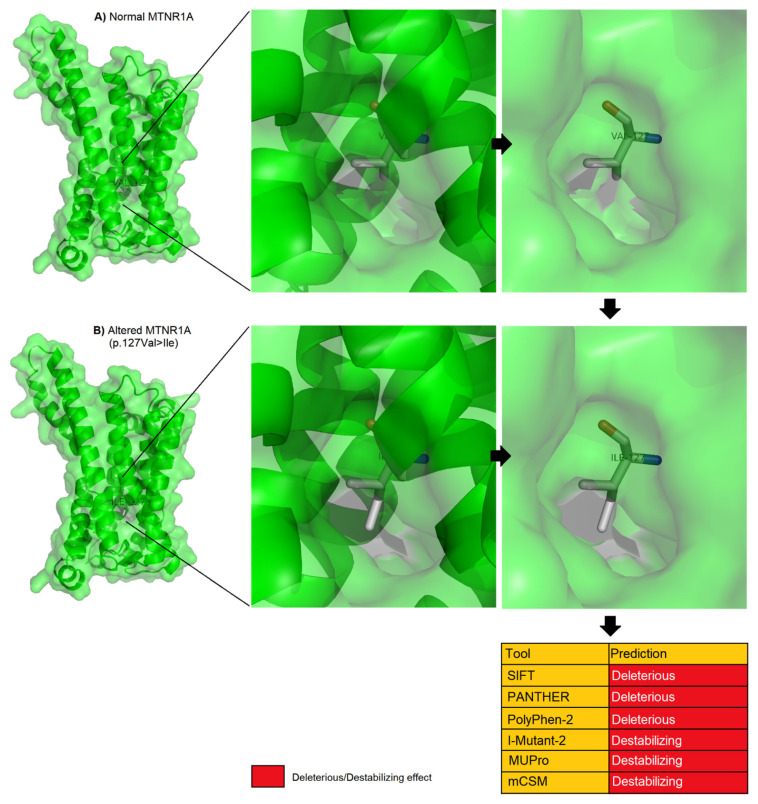
The in-silico analysis of the deleterious effects of the observed missense p.127Val>Ile variant in the MTNR1A. The 3D structure is shown as cartoons encrypted within a transparent surface.

**Table 1:** Identification code of the variant, position in the current assembly, and amino acid changes

Alias	Uploaded variant	Location	Amino acid change	MTNR1A region	Uploaded allele
snp1	g.15571529G>C	26:15571529	Arg/Gly	Exon2	G/C
snp2	rs1087815963G>C	26:15571508	Val/Ile	Exon2	C/T
snp3	rs419680097G>T	26:15571482	None	Exon2	C/A
snp4	rs1091928580G>C	26:15571480	Gly/Val	Exon2	C/A
snp5	g.15571439A>C	26:15571439	Gly/*	Exon2	A/C
snp6	g.15571438G>A	26:15571438	Thr/Ile	Exon2	G/A
snp7	g.15571433G>A	26:15571433	Arg/Cys	Exon2	G/A
snp8	rs1088397747	26:15571413	None	Exon2	C/T
snp9	rs406779174	26:15571329	None	Exon2	G/A
snp10	rs430181568	26:15571323	None	Exon2	C/T
snp11	rs420819884	26:15571260	None	Exon2	T/C
snp12	rs407388227	26:15571229	Val/Ile	Exon2	C/T
snp13	rs427019119	26:15571152	None	Exon2	C/T
snp14	rs417800445	26:15571134	None	Exon2	C/T
snp15	rs588561468	26:15571104	None	Exon2	G/A
snp16	rs429718221	26:15571044	None	Exon2	G/A
snp17	rs416266900	26:15571042	Ala/Asp	Exon2	G/T
snp18	rs403212791	26:15570842	Arg/Cys	Exon2	G/A
snp19	g.15570837C>A	26:15570837	Lys/Asn	Exon2	C/A

**Table 2:** Frequencies of alleles and genotypes of the *MTNR1A* gene in thin-tailed Indonesian ewes

SNP number	dbSNPs	Genotype	Genotype frequency	PIC	HWE (p-value)	Allele	Allele frequency
snp1	g.15571529	CC	0	0.428	0.002	C	0.309
		GC	0.617			G	0.691
		GG	0.383				
snp2	rs1087815963	CC	0	0.148	0.611	C	0.074
		GC	0.149			G	0.926
		GG	0.851				
snp3	rs419680097	GG	0.830	0.214	0.00044	G	0.883
		GT	0.106			T	0.117
		TT	0.064				
snp4	rs1091928580	CC	0	0.217	0.339	C	0.128
		GC	0.255			G	0.872
		GG	0.745				
snp5	g.15571439	AA	0.830	0.160	0.552	A	0.915
		CA	0.170			C	0.085
		CC	0				
snp6	g.15571438	AA	0.043	0.370	0.566	A	0.245
		AG	0.404			G	0.755
		GG	0.553				
snp7	g.15571433	AA	0	0.251	0.210	A	0.160
		AG	0.319			G	0.840
		GG	0.681				
snp8	rs1088397747	GG	0.809	0.183	0.495	G	0.904
		GT	0.191			T	0.096
		TT	0				
snp9	rs406779174	CC	0.447	0.466	0.212	C	0.639
		CT	0.383			T	0.361
		TT	0.170				
snp10	rs430181568	AA	0.043	0.217	0.082	A	0.128
		AG	0.170			G	0.872
		GG	0.787				
snp11	rs420819884	AA	0	0.021	1.00	A	0.010
		AG	0.021			G	0.99
		GG	0.979				
snp12	rs407388227	AA	0.149	0.278	0.0000	A	0.170
		AG	0.043			G	0.830
		GA	0.809				
snp13	rs427019119	AA	0.255	0.375	0.0000	A	0.260
		AG	0			G	0.740
		GG	0.745				
snp14	rs417800445	AA	0.234	0.375	0.0000	A	0.260
		AG	0.043			G	0.740
		GG	0.723				
snp15	rs588561468	CC	0.936	0.095	0.0000	C	0.970
		CT	0			T	0.063
		TT	0.064				
snp16	rs429718221	CC	0.531	0.493	0.0000	C	0.553
		CT	0.043			T	0.447
		TT	0.426				
snp17	rs416266900	AA	0.234	0.375	0.0000	A	0.255
		AC	0.043			C	0.745
		CC	0.723				
snp18	rs403212791	CC	0.617	0.365	0.230	C	0.766
		CT	0.298			T	0.234
		TT	0.85				
snp19	g.15570837	AA	0.064	0.095	0.0000	A	0.063
		AC	0			C	0.937
		CC	0.936				

SNP, single nucleotide polymorphism; PIC, polymorphism information content; HWE, Hardy–Weinberg equilibrium.

**Table 3:** Least squares mean and standard error for litter size of different genotypes of the *MTNR1A* gene in Thin-tailed Indonesian ewes

SNP number	dbSNPs	Genotype	LSmeans SNP
snp1	g.15571529	GC (19)	1.76±0.577
		GG (28)	1.89±0.832
snp2	rs1087815963	GC (7)	2.57±0.787^a^
		GG (40)	1.68±0.572^b^
snp3	rs419680097	GG (39)	1.82±0.721
		GT (5)	1.60±0.548
		TT (3)	2.00±0.000
snp4	rs1091928580	GC (12)	1.58±0.515
		GG (35)	1.89±0.718
snp5	g.15571439	AA (39)	1.77±0.667
		CA (8)	2.00±0.756
snp6	g.15571438	AA (2)	2.50±0.707
		AG (19)	1.68±0.671
		GG (26)	1.85±0.675
snp7	g.15571433	AG (15)	1.60±0.737
		GG (32)	1.91±0.641
snp8	rs1088397747	GG (38)	1.79±0.664
		GT (9)	1.89±0.782
snp9	rs406779174	CC (21)	1.81±0.750
		CT (18)	1.78±0.647
		TT (8)	1.88±0.641
snp10	rs430181568	AA (2)	2.00±0.000
		AG (8)	1.63±0.518
		GG (37)	1.84±0.727
snp11	rs420819884	AG (1)	4.00^a^
		GG (46)	1.76±0.603^b^
snp12	rs407388227	AA (7)	1.71±0.488
		AG (2)	1.00±0.000
		GA (38)	1.87±0.704
snp13	rs427019119	AA (11)	1.82±0.751
		GG (36)	1.81±0.668
snp14	rs417800445	AA (11)	1.82±0.751
		AG (2)	2.00±1.414
		GG (34)	1.79±0.641
snp15	rs588561468	CC (44)	1.82±0.691
		TT (3)	1.67±0.577
snp16	rs429718221	CC (25)	1.84±0.688
		CT (2)	2.00±1.414
		TT (20)	1.75±0.639
snp17	rs416266900	AA (11)	1.82±0.751
		AC (2)	2.00±01.414
		CC (34)	1.79±0.641
snp18	rs403212791	CC (29)	1.69±0.604
		CT (14)	2.07±0.829
		TT (4)	1.75±0.500
snp19	g.15570837	AA (3)	2.00±1.00
		CC (44)	1.80±0.668

SNP, single nucleotide polymorphism; LSmean, least squares mean.

a,b Values with different superscripts within the same column differ significantly (p<0.05).

**Table 4:** Estimated linkage disequilibrium for the 19 single nucleotide polymorphisms identified in the *MTNR1A* gene

Block	D′	r^2^
Block 1		
g.15571529/ rs1087815963	0.354	0.005
g.15571529/ rs419680097	1	0.059
rs1087815963/ rs419680097	1	0.011
Block 2		
g.15571439 / g.15571438	0.553	0.088
Block 3		
rs406779174/ rs430181568	1	0.083
rs406779174/ rs420819884	1	0.006
rs430181568/ rs420819884	1	0.002
Block 4		
rs407388227/ rs427019119	0.755	0.04
rs407388227/ rs417800445	1	0.07
rs427019119/ rs417800445	0.888	0.789
Block 5		
rs588561468/ rs429718221	1	0.084
rs588561468/ rs416266900	1	0.023
rs588561468/ rs403212791	1	0.021
rs429718221/ rs416266900	1	0.424
rs429718221/ rs403212791	0.895	0.198
rs416266900/ rs403212791	0.804	0.068

**Table 5:** Estimates of haplotype frequencies and association analysis of *MTNR1A* gene haplotypes and litter size in Thin-tailed Indonesian sheep^1)^

Block^2)^	Haplotype	Number of samples	Haplotype frequencies	Litter size (mean±standard deviation)	p-value
1	H1 (GGG)	44	0.503	1.79±0.701^b^	<0.05
	H2 (CGG)	29	0.305	1.75±0.576^b^	
	H3 (GGT)	7	0.117	1.71±0.487^ab^	
	H4 (GCG)	6	0.071	2.33±0.516^a^	
2	H1 (AG)	44	0.727	1.77±0.677	0.82
	H2 (AA)	21	0.188	1.76±0.700	
	H3 (CA)	4	0.056	2.00±1.154	
3	H1 (CGG)	37	0.5	1.78±0.712	0.85
	H2 (TGG)	26	0.362	1.81±0.634	
	H3 (CAG)	9	0.128	1.67±0.500	
4	H1 (GGG)	28	0.564	1.86±0.705	0.70
	H2 (GAA)	11	0.234	1.82±0.751	
	H3 (AGG)	8	0.160	1.63±0.518	
5	H1 (CCCC)	21	0.330	1.86±0.727	0.84
	H2 (CTAC)	13	0.244	1.85±0.801	
	H3 (CCCT)	17	0.223	2.00±0.791	
	H4 (CTCC)	9	0.128	1.67±0.707	
	H5(TTCC)	3	0.064	1.67±0.577	

1) Haplotypes with a frequency below 0.01 are not shown.

2) Block1, snp1-snp2-snp3; Block2, snp5-snp6; Block3, snp9-snp10-snp11; Block4, snp12-snp13-snp14; Block 5, snp15-snp16-snp17-snp18-snp19 -snp_25-snp_26-snp_28-snp_29-snp_30-snp_32-snp_33-snp_34.

## SUPPLEMENTARY MATERIAL

**Supplementary File S1.** Ramachandran plot output data for the generated MTNR1A protein.

**Supplementary File S2.** ProSA Web plot output data for the generated MTNR1A protein.
