# Supplementary figures and images for: A novel p.127Val>Ile single nucleotide polymorphism in the MTNR1A gene and its relation to litter size in Thin-tailed Indonesian ewes
Source: Anim Biosci. 2024 Jun 25;38(2):209–22. doi: 10.5713/ab.24.0187 (PMC11725752; doi:10.5713/ab.24.0187)

Supplementary File 1. Ramachandran plot output data for the generated MTNR1A protein

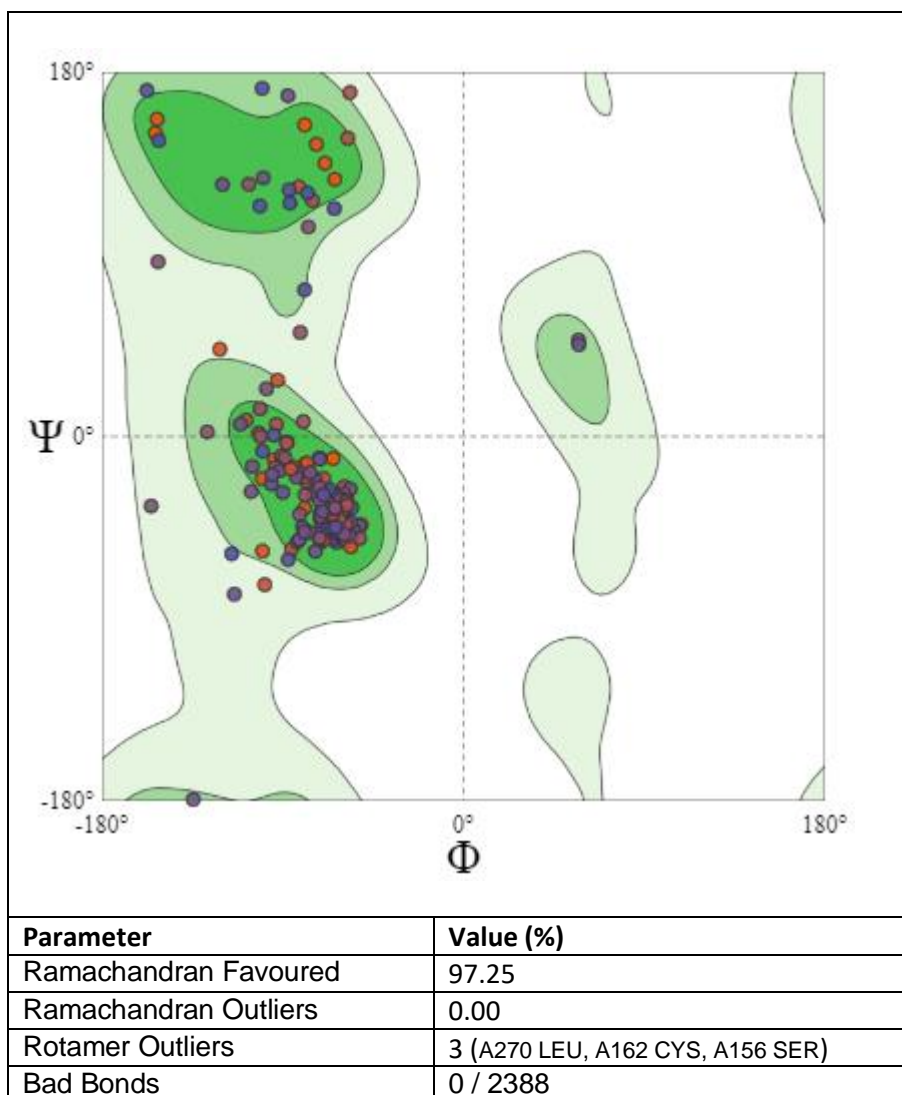

Supplement: Supplementary file 1 [file ab-24-0187-Supplementary-Fig-1.pdf]

Supplementary File 2. ProSA Web plot output data for the generated MTNR1A protein

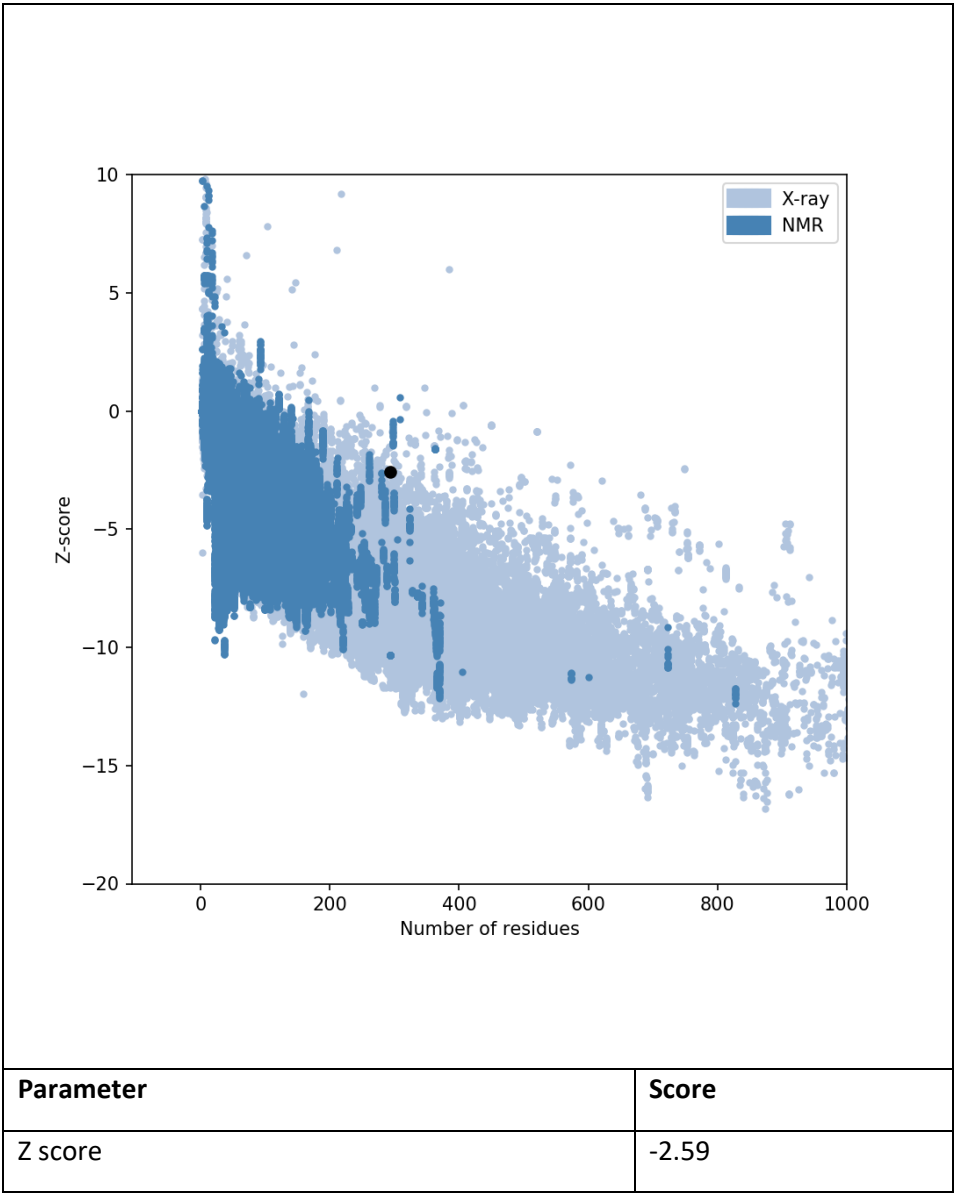

Supplement: Supplementary file 2 [file ab-24-0187-Supplementary-Fig-2.pdf]
